# A novel surgical approach and technique and short-term clinical efficacy for the treatment of proximal humerus fractures with the combined use of medial anatomical locking plate fixation and minimally invasive lateral locking plate fixation

**DOI:** 10.1186/s13018-020-02094-7

**Published:** 2021-01-09

**Authors:** Fu Wang, Yan Wang, Jinye Dong, Yu He, Lianxin Li, Fanxiao Liu, Jinlei Dong

**Affiliations:** 1 Department of Orthopedics, Shandong Provincial Hospital Affiliated to Shandong First Medical University, Shandong Provincial Hospital Affiliated to Shandong University, 324 Jing Wu Road, Jinan, 250021 People’s Republic of China; 2 Medical Laboratory Diagnosis Center, Jinan Central Hospital, 105 Jiefang Road, Jinan, 250013 People’s Republic of China; 3 Department of Ultrasound, Weifang People’s Hospital, Weifang, 261041 Shandong People’s Republic of China; 4 Department of Plastic Surgery, Plastic Surgery Hospital, Chinese Academy of Medical Sciences & Peking Union Medical College, 33 Badachu Road, Shijingshan District, Beijing, 100144 People’s Republic of China

**Keywords:** Proximal humerus fractures, Medial surgical approach, Locking plate, Technique, Anatomy, Minimally invasive lateral fixation

## Abstract

**Background and hypothesis:**

The typical anterolateral approach is widely used to treat proximal humerus fractures with lateral locking fixation. However, lateral fixation cannot completely avoid medial reduction loss and varus deformity especially in the cases of an unstable medial column. We present a novel medial surgical approach and technique together with a minimally invasive lateral locking plate to fix proximal humerus fractures with an unstable medial column.

**Materials and methods:**

We performed an anatomical study and reported 8 cases of proximal humerus fractures with unstable medial columns treated with plate fixation through a minimally invasive anterolateral approach and medial approach. All surgeries were performed by the same single surgeon. Patients were followed clinically and radiographically at 1, 3, 6, and 12 months postoperatively.

**Results:**

There was a safe region located at the medial part of the proximal humerus just beneath the articular surface. An anatomical medial locking proximal humerus plate could be placed in the medial column and did not affect the axillary nerve, blood supply of the humeral head, or stability of the shoulder joint. Successful fracture healing was achieved in all 8 cases. The function and range of motion of the shoulder joint were satisfactory 24 months postoperatively, with an average Constant score (CS) of 82.8. No reduction loss (≥ 10° in any direction), screw cutout, nonunion, or deep infection occurred.

**Conclusions:**

The combined application of medial anatomical locking plate fixation and minimally invasive lateral locking plate fixation is effective in maintaining operative reduction and preventing varus collapse and implant failure in proximal humerus fractures with an unstable medial column.

## Introduction

Proximal humerus fractures are relatively common, accounting for approximately 5-6% of fractures in adults [1, 2]. In 1970, Neer introduced a classification of fractures of the proximal humerus, which is divided into 2-, 3-, or 4-fragment types. The Neer classification provides important information concerning the risk of damage to the humeral head vasculature, which increases with the number of bone fragments of the damaged head. This information provides great prognostic value [3].

The treatment of proximal humerus fractures may be challenging and must be considered carefully. Open reduction and internal fixation is the optimal treatment for complex fractures to maximize functional recovery [4]. Lateral locking plate fixation through the anterolateral approach is typically used for the treatment of complex proximal humerus fractures [5]. Unfortunately, loss of medial cortical support has been identified as a key risk factor for humeral head subsidence with loss of reduction and intra-articular screw perforation [6]. Therefore, reconstruction of the medial column can provide cortical support for the humeral head, which may be useful to avoid those complications. For fractures without stable medial columns, medial support can be restored by anatomical reduction to reduce the risk of fixation failure. Several researchers have revealed that the mechanical support of the medial column provided by additional medial support screws can be of great importance for establishing a stable construct [7, 8]. However, Bai et al. [9] reported that the supporting effect of calcar screws was not notable in varus (20°) humeral heads fixed with locking plates, concluding that direct medial support may be a more effective strategy. Hence, we considered that direct medial support and fixation may be more effective in providing dual-column support and antirotational stability. In our previous finite element study and biomechanical evaluation analysis of this novel medial fixation technique [10, 11], we found that the medial plate could disperse the stress on the lateral plate, which may reduce the risk of implant failure by transferring the loads through both the lateral and medial columns.

In the present study, we performed an anatomical study and reported 8 cases of proximal humerus fractures with unstable medial columns treated with plate fixation through a minimally invasive anterolateral approach and medial approach with a mean follow-up of 12 months. This study may provide valuable information to surgeons towards restoring medial stability in order to provide cortical support for the humeral head.

## Materials and methods

All experimental procedures were approved by the Shandong Provincial Hospital Ethics Committee. All aspects of this study were conducted with adherence to the current version of the Declaration of Helsinki, the guidelines established by the International Conference on Harmonization of Good Clinical Practice, and the laws of China. All participants signed informed consent forms before enrollment.

### Anatomical study

**Fig. 1:**
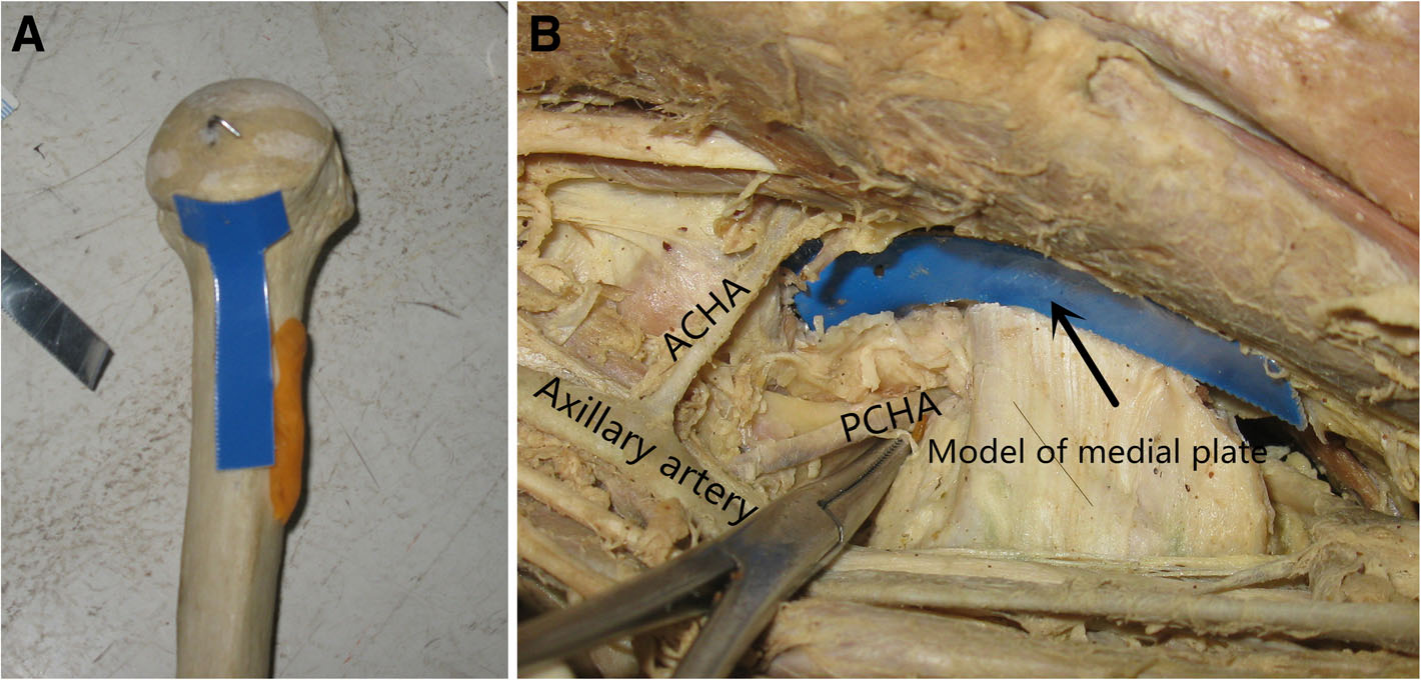
Anatomical study and fixation design. **a** The metal model of the plate placed on the bone. **b** The region between the ACHA and PCHA

**Fig. 2:**
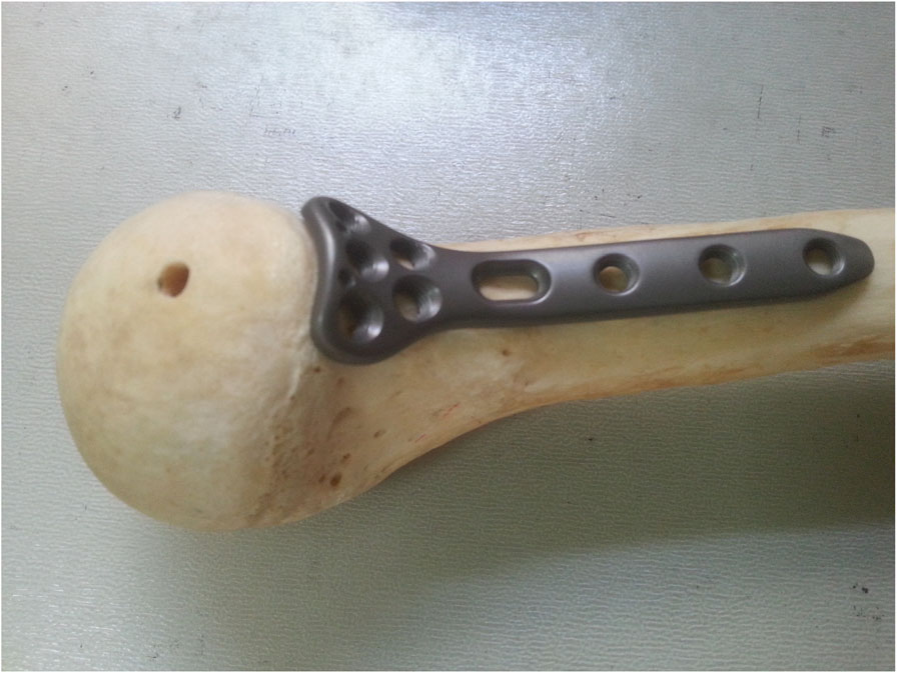
An anatomical locking plate designed for medial fixation of proximal humerus fractures

Dissection was performed in 6 bodies donated to science, which included 4 males and 2 females. In each body, both the left and right shoulders were carefully dissected. The upper limbs were placed with lateral rotation of the shoulder joint and flexion of the elbow joint. After incision of subcutaneous tissue, the biceps brachii and coracobrachialis muscles were exposed, and the two muscles together with the musculocutaneous nerves between them were pulled laterally. Then, the brachial blood vessels and the rest of the brachial plexus were identified and pulled medially. We could see the conjoined tendon of the latissimus dorsi and teres major muscles and we paid attention to protecting the anterior and posterior circumflex vessels above the conjoined tendon. The tendon was marked with sutures and then cut. The conjoined tendon then shrank medially. Dissection was continued proximally from the joint tendon insertion. After those steps, the lower part of the shoulder capsule, anterior circumflex humeral artery (ACHA), posterior circumflex humeral artery (PCHA), and axillary nerve were all visible. The nerve and blood vessels were protected; then, the capsule was opened directly in the longitudinal direction (Figs. 1 and 2).

The following data were measured and observed: (1) the position and diameter of the anterior and posterior circumflex brachial arteries starting from the brachial artery, (2) the distance from the origin of the anterior and posterior circumflex brachial arteries to the subglenoid nodule, (3) the distance between the anterior and posterior circumflex brachial arteries at the level of the lower edge of the humeral head, and (4) whether there was a communicating branch between the anterior and posterior circumflex brachial arteries.

### Patients, surgical techniques, and clinical results

Between November 2014 and February 2017, eight cases of proximal humerus fractures with unstable medial columns were treated operatively. The characteristics of the 8 cases are presented in Table 1. According to the Neer classification [3], 4 were three-part fractures, and 4 were four-part fractures.

**Table 1:** The demographic and clinical characteristics and final functional results in 8 cases

Cases	Gender	Age, years	Side	Mechanism of injury	Pattern of fracture (Neer system)	Operative time (min)	FU (months)	Complications	Constant score of 24 months post-operation
1	F	62	Left	MVC	3-part	205	24	None	86
2	F	45	Left	MVC	3-part	195	24	None	84
3	F	56	Right	Fall	4-part	172	12	None	83
4	M	62	Right	MVC	4-part	170	23	None	82
5	M	62	Left	Fall	4-part	165	24	None	80
6	F	38	Left	MVC	3-part	175	14	None	82
7	M	65	Left	MVC	3-part	168	12	None	82
8	M	43	Right	MVC	4-part	165	12	None	83
Mean		54.1				176.9	18.1		82.8

*FU* follow-up, *MVC* motor vehicle collision

**Fig. 3:**
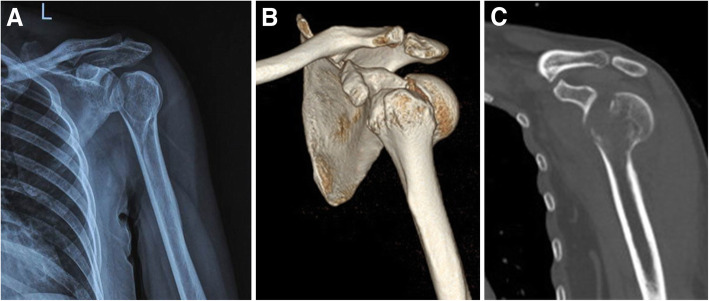
Case 2. A 45-year-old woman. AP X-ray (**a**) and 3-D CT (**b**, **c**) of the patient’s left shoulder immediately after injury demonstrating a fracture of the proximal humerus

**Fig. 4:**
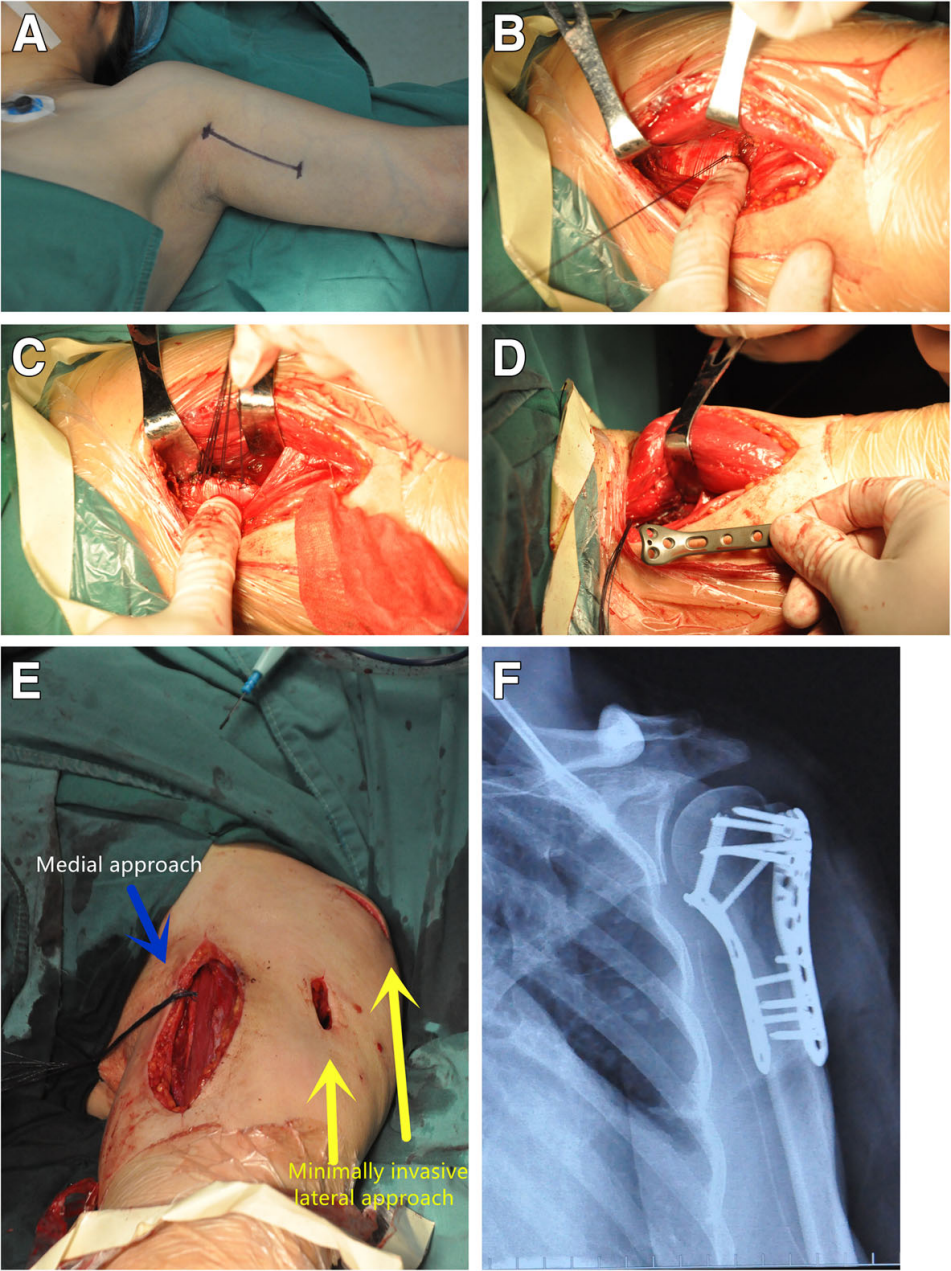
Intraoperative photograph of medial fixation of proximal humerus fractures. **a** The medial incision was marked with a pen before the operation. **b** The conjoined tendon of the latissimus dorsi and teres major muscles was identified. **c** The conjoined tendon was marked with suture traction and cut. **d** A K-wire was inserted through the incision for temporary fixation, and then a locking plate of appropriate length was inserted along the medial aspect of the humerus. **e** A PHILOS plate was used through a minimally invasive anterolateral approach. **f** Immediate postoperative radiographs

As shown in Figs. 3 and 4, a 45-year-old woman suffered a three-part comminuted proximal humerus fracture. General anesthesia was selected and the patient was placed in the supine position. The medial operation was applied first, and the arm was externally rotated, abducted with elbow flexion. Our medial approach involves several procedures as described herein (Fig. 4). The incision began proximally from the front end of the armpit (junction of the biceps brachii muscle and pectoralis major muscle). It was then extended towards the medial epicondyle of the humerus with a length of approximately 12 cm. The next steps were as mentioned in the section on the anatomical study and fixation design. After opening the lower part of the shoulder capsule longitudinally, a fracture fragment beneath the humeral head was visible. It should be noted that the exposed fore-and-aft region should not exceed 3 cm when opening the medial capsule longitudinally to avoid injury to the ACHA and PCHA. Care should also be taken to avoid damaging the glenohumeral ligament (GHL).

Fracture reduction was achieved with rotation and traction of the arm. For temporary fixation, a K-wire was inserted through the medial incision and another K-wire was percutaneously inserted laterally. Then, the newly designed anatomical locking plate (Waston, China) with an appropriate length was inserted along the medial aspect of the humerus. First, a cortex screw was fixed on the slotted sliding hole, so the plate’s location was appropriately adjusted in the distal and proximal directions. Then, 3 locking screws were inserted into the humeral head and 2 locking screws were inserted into the distal portion of the plate (Fig. 4f). Finally, the cortex screw and two K-wires were removed.

Then, a two-incision minimally invasive anterolateral approach was used to fix the lateral fracture of the proximal humerus as previously reported [12]. A locking plate of appropriate length was inserted from the proximal incision to the distal portion of the fracture along the lateral aspect of the humerus. Finally, the previously cut conjoined tendon of the latissimus dorsi and teres major muscles was restored with suturing.

The patient underwent closure over a small suction drain and was immobilized in a sling for the first 10 days postoperatively. Physical therapy was started with gentle Codman exercises and active-assisted range of motion within the first 2 weeks postoperatively. Gentle resistive exercises with un-restricted passive motion were initiated 6 weeks postoperatively.

## Results

Through measurement and observation, we found that (1) the ACHA and PCHA originated from almost the same level of the axillary artery and the mean distance between the origins of the ACHA and PCHA was 3.1 mm (0.2 to 4.2 mm); (2) the mean diameter of the ACHA was 0.9 mm (0.7 to 1.2 mm) and the mean diameter of the PCHA was 1.9 mm (1.6 to 2.2 mm); (3) the mean distance from the origin of ACHA and PCHA to the infraglenoid tubercle (PI) was 27.1 mm (25.1 to 30.8 mm); (4) no communicating branches were found between the PCHA and ACHA in all specimens; and (5) the lower part of the shoulder capsule was loose, so the longitudinal incision did not affect the stability of the shoulder joint.

The anatomical results above reveal that (1) the medial approach can provide good exposure to the medial column of the proximal humerus and (2) there is a safe region located at the medial part of the proximal humerus just beneath the articular surface. As shown in Fig. 1, a blue metal model with the same size as the designed anatomical medial locking plate can be placed in that region and will not affect the axillary nerve, blood supply of the humeral head, or stability of the shoulder joint.

According to the anatomical research results described above, an anatomical locking proximal humerus plate was designed and manufactured (Waston, China) (Fig. 2), and it features an arrangement of 3 or 5 locking screw holes for fixation in the humeral head, a slotted sliding hole for a K-wire or a nonlocked shaft screw, and 2–4 locking compression holes in the distal part for fixation to the humeral shaft.

Between November 2014 and February 2017, 8 (4 women and 4 men; mean age 54.1 years; age range, 38–65 years; 3 right and 5 left shoulders) with medial cortical deficiency were treated with medial anatomical locking plate fixation and minimally invasive lateral locking plate fixation and were retrospectively enrolled in this study. All surgeries were performed by the same single surgeon (Fu Wang) with the average surgical duration of 176.9 min. The patients were followed clinically and radiographically 1, 3, 6, and 12 months postoperatively. All eight patients were followed for a period of 35–84 months (average 38).

**Fig. 5:**
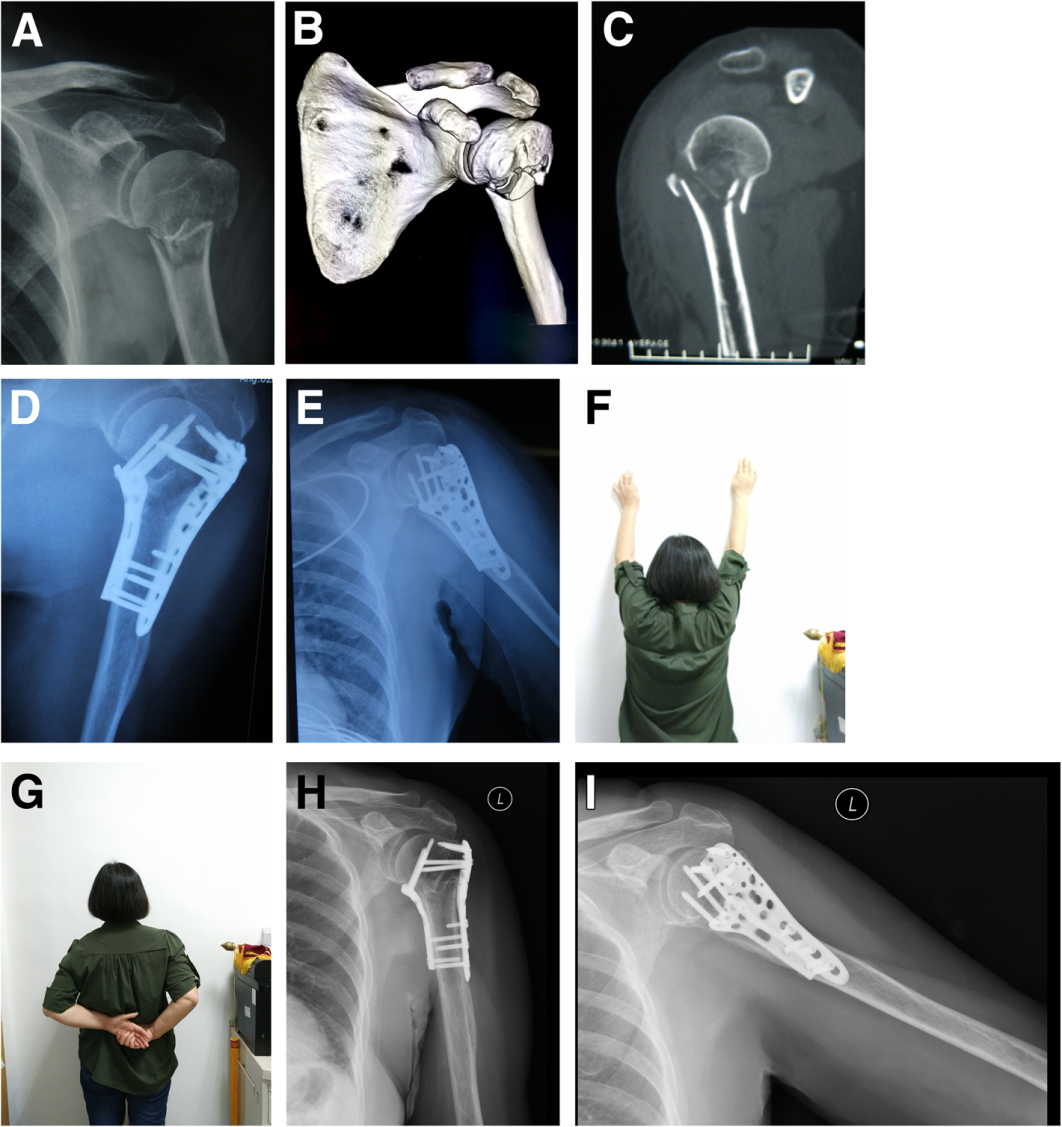
Case 1. A 62-year-old woman. AP X-ray (**a**) and 3-D CT (**b**, **c**) of the patient’s left shoulder immediately after injury demonstrating a fracture of the proximal humerus. Immediate postoperative anteroposterior (**d**) and lateral (**e**) radiographs. Clinical (**f**, **g**) and radiological (**h**, **i**) results 12 months postoperatively (Constant score 84)

Successful fracture healing was achieved in all 8 cases (Table 1). The function and range of motion of the shoulder joint were satisfactory 24 months postoperatively with an average Constant score [13] (CS) of 82.8. No reduction loss (≥ 10° in any direction), screw cutout, nonunion, or deep infection occurred. In one case, superficial infection of the medial incision occurred after the operation. After the regular dressing changes, the infection recovered completely 1 month after operation. In addition, no avascular head necrosis was found any of the 8 cases 24 months postoperatively (Fig. 5).

## Discussion

The typical anterolateral approach (including the minimally invasive anterolateral approach) is the working horse for the surgical treatment of proximal humerus fractures with locking fixation [13, 14]. Despite the biomechanical superiority and clinical availability of this treatment approach, several studies have documented reduction loss and varus deformity in proximal humeral fractures treated with locking plates. A common pattern of failure is the increased varus displacement of the humeral head, leading to secondary screw penetration or plate breakage [13, 15–17]. Jung et al. [18] revealed that insufficient medial support was an independent risk factor for reduction loss in proximal humerus fractures (*p* < 0.01). Maier et al. [19] found that short or absent metaphyseal head extension and disruption of medial periosteal hinge reliably predict posttraumatic humeral head ischemia. Hertel et al. [20] identified that the reliable predictors of ischemia were short (< 8 mm) posteromedial metaphyseal extension of the head fragment and disruption of the medial hinge with displacement of > 2 mm. Krappinger et al. [21] pointed out that local bone mineral density, restoration of the medial column, nonanatomic reduction, and age were significant predictors of fixation failure (*P* < 0.01).

To resolve medial reduction loss and varus deformity, two points were presented [22] (1) using calcar screws to provide angular stability, facilitating the maintenance of humeral head reduction, and (2) recovering the continuity of the medial cortex to provide cortical support for the humeral head. The use of medial support screws, screw-tip augmentation with bone cement and bone grafts are currently the most frequently used and assessed tools to increase the stability of the locking plate complex [23–25]. However, their functional outcomes were unpredictable and inconsistent [26, 27].

To achieve medial stability, Wanner et al. suggested a technique with the additional use of one third of tubular plates positioned ventrally and at a right angle to the lateral adjusted standard plate [28]. However, this technique resulted in a decrease in biomechanical stability compared to fixation with locking plate systems [29]. The ventrally inserted plate may damage the blood supply of the arcuate artery [30]. Park and Ko [31] showed that the use of additional medial buttress plate fixation in proximal humeral fractures with unstable medial column restoration after lateral locking compression plate fixation provided strong support for the medial column. In this study, we used an anatomical locking plate to fix the medial column, which has two advantages. First, compared with those of anatomical plates, the locking mechanism and interference with the mechanical stability of the plate may be changed by plate bending during the operation. Furthermore, no intraoperative plate bending will reduce the operation time.

In this study, the combined application of minimally invasive lateral locking plate fixation and medial locking plate fixation was performed, which could provide a stable dual-column buttress for the treatment of proximal humerus fractures with unstable medial columns or osteoporosis. A potential problem of the double-plating technique seems to be the medial plate, which might damage the blood supply of the humeral head by compressing the branch of the circumflex artery [32]. No humeral head necrosis was observed in this study, which suggests that blood supply is sufficient for survival of the humeral head. The PCHA and ACHA have been proven to be crucial for the blood supply of the humeral head [33]. To avoid posttraumatic humeral head ischemia, the PCHA and ACAH were protected carefully during the operation in this study. According to a previous study and our anatomical study, there are no communicative branches between the PCHA and ACHA. In addition, beneath the humeral head, the distance between the PCHA and ACAH is approximately 25–30 mm. Therefore, both the PCHA and ACAH can be avoided by using this interval plane, and there is enough space to place the plate at the medial column. Moreover, a recent case report by Gerber et al. [34] underlined the clinical relevance of intraosseous blood supply by anastomoses of the deep brachial artery because avascular necrosis of the humeral head was absent after posttraumatic rupture of the anterior and posterior humeral circumflex arteries. It should be emphasized that the surgeon must take care to avoid injury to the GHL. In addition, based on our measurement and experience, the exposed fore-and-aft region should not exceed 3 cm when the medial capsule is opened longitudinally to avoid injury to the ACHA and PCHA. However, we should admit that the medial approach remains difficult to learn because of the relatively complex anatomical structure and spatial relationships.

According to our observations, it is not complicated to place the medial column plate, and there is limited risk of injury to the nerve and vessels while fixing the plate and screws. Appropriate plating was needed for fixation of the medial column because of its relatively complex anatomic structure. The proximal portion of the plate should be plate between PCHA and ACHA, and its width should be limited to less than 25 mm to avoid those two vessels. In our cases, an anatomical locking plate was designed and used, and 3 locking screws were inserted into the humeral head with 2 locking screws inserted into the humeral shaft.

In this clinical trial, we describe not a completely new treatment strategy but a new surgical technique and the use of a new implant, to restore medial cortical support in proximal humeral fractures. In this regard, we observed a mean Constant score of 82.8 24 months after the operation. Thus, the clinical results from our patient population appear to be satisfactory, and we consider our results relevant despite the small number of included patients.

This study has several limitations. First, the sample size was small, which may not have been sufficient for accurately assessing the efficacy of this technique. Second, the follow-up period was short, and the biomechanics of this medial locking plating have not been studied. Third, there was no control group to permit a head-to-head comparison with other surgical techniques.

## Conclusion

The medial approach is a safe approach with limited muscle detachment, providing good exposure to the medial column of the proximal humerus. Through this approach and technique, stable medial plate fixation recovering the continuity of the medial cortex may be useful to avoid humeral head subsidence. Combined application of medial anatomical locking plate fixation and minimally invasive lateral locking plate fixation is effective in maintaining operative reduction and preventing varus collapse and implant failure in proximal humerus fractures with an unstable medial column.

## Data Availability

All data analyzed during this study are included in this published article.

## Abbreviations

ACHA: Anterior circumflex humeral artery
CS: Constant score
GHL: Glenohumeral ligament
PCHA: Posterior circumflex humeral artery
PI: Infraglenoid tubercle
